# Meropenem in combination with baicalein exhibits synergism against extensively drug resistant and pan-drug-resistant *Acinetobacter baumannii* clinical isolates *in vitro*

**DOI:** 10.1093/femspd/ftad007

**Published:** 2023-04-29

**Authors:** Mümtaz Güran, Kadir Çakıral, Kerem Teralı, Tülay Kandemir, Gizem Şanlıtürk, Melda Meral Öcal, Toğrul Nagiyev, Fatih Köksal

**Affiliations:** Department of Medical Microbiology, Faculty of Medicine, Eastern Mediterranean University, 99628 Famagusta, N. Cyprus via Mersin 10, Turkey; Department of Medical Microbiology, Faculty of Medicine, Eastern Mediterranean University, 99628 Famagusta, N. Cyprus via Mersin 10, Turkey; Department of Chemistry, Faculty of Arts and Sciences, Eastern Mediterranean University, 99628 Famagusta, N. Cyprus via Mersin 10, Turkey; Department of Medical Biochemistry, Faculty of Medicine, Cyprus International University, 99258 N. Cyprus via Mersin 10, Turkey; Department of Medical Microbiology, Faculty of Medicine, Çukurova University, 1380 Adana, Turkey; Department of Medical Microbiology, Faculty of Medicine, Eastern Mediterranean University, 99628 Famagusta, N. Cyprus via Mersin 10, Turkey; Department of Chemistry, Faculty of Arts and Sciences, Eastern Mediterranean University, 99628 Famagusta, N. Cyprus via Mersin 10, Turkey; Department of Medical Microbiology, Faculty of Medicine, Çukurova University, 1380 Adana, Turkey; Department of Biotechnology, Faculty of Science, The Campus of Çiftlikköy, Mersin University, 33343 Yenişehir, Mersin, Turkey; Department of Medical Microbiology, Faculty of Medicine, Çukurova University, 1380 Adana, Turkey; Department of Medical Microbiology, Faculty of Medicine, Çukurova University, 1380 Adana, Turkey

**Keywords:** *Acinetobacter baumannii*, baicalein, carbapenem, synergy

## Abstract

Several studies have demonstrated that the effectiveness of carbapenems against drug-resistant *Acinetobacter baumannii* infections has been decreasing. Combination therapy with two or more drugs is currently under investigation to overcome the emerging resistance against carbapenems. In this study, we tested the possible synergistic interactions of a potent antibacterial flavonoid, baicalein, with meropenem to illustrate this duo’s antibacterial and antibiofilm effects on 15 extensively drug resistant or pan-drug-resistant (XDR/PDR) *A. baumannii* clinical isolates *in vitro*. Isolates included in the study were identified with MALDI-TOF MS, and antibiotic resistance patterns were studied according to EUCAST protocols. Carbapenem resistance was confirmed with the modified Hodge test, and resistance genes were also analyzed with genotypical methods. Then, checkerboard and time-kill assays were performed to analyze antibacterial synergism. Additionally, a biofilm inhibition assay was performed for screening the antibiofilm activity. To provide structural and mechanistic insights into baicalein action, protein–ligand docking, and interaction profiling calculations were conducted. Our study shed light on the remarkable potential of the baicalein–meropenem combination, since either synergistic or additive antibacterial activity was observed against every XDR/PDR *A. baumannii* strain in question. Furthermore, the baicalein–meropenem combination displayed significantly better antibiofilm activity in contrast to standalone use. *In silico* studies predicted that these positive effects arose from inhibition by baicalein of *A. baumannii* beta-lactamases and/or penicillin-binding proteins. Overall, our findings highlight the prospective potential benefits of baicalein in combination with meropenem for the treatment of carbapenem-resistant *A. baumannii* infections.

## Teaser

Remarkable therapeutic potential of meropenem–baicalein combination is presented in this *in vitro* study.

## Introduction

With its exceptional capacity to cause chronic and nosocomial infections *Acinetobacter baumannii* is a bacterium posing severe threat to public health. Even last-resort antibiotics and their combinations are becoming ineffective with an increasing pace (Assimakopoulos et al. 2019), placing this pathogen among the most critically resistant bacteria according to the World Health Organization (WHO 2017) and stressing the immediacy of the need for new treatment strategies.

Combined use of carbapenems or fluoroquinolones has gained popularity instead of using the antibiotic alone especially against multidrug-resistant (MDR), extensively drug resistant (XDR; MDR plus resistance to carbapenems), and pan-drug-resistant (PDR; XDR plus resistance to polymyxins) *A. baumannii* strains (Karaiskos and Giamarellou 2014, Karakonstantis et al. 2021, Huang et al. 2022). Furthermore, antimicrobial monotherapy is not suggested in infections caused by colistin-resistant *A. baumannii* strains in clinical practice (Hong et al. 2016). At this point, alternative novel strategies where antibiotics are combined with antimicrobially efficient polyphenols, peptides, and phages have emerged and gained momentum over the years against the above-mentioned resistant *A. baumannii* strains (Jubair et al. 2021, Caraway et al. 2022, Meng et al. 2022, Sisakhtpour et al. 2022).

Baicalein (BCL) (5,6,7-trihydroxy-2-phenylchromen-4-one) is a trihydroxyflavone obtained from the root extracts of *Scutellaria baicalensis*. Previous investigations have proposed that BCL possess antimicrobial activity in addition to many other health benefits such as antioxidant, anti-inflammatory, antihypertensive, and anticancer properties (Wan et al. 2018, Chen et al. 2021). Furthermore, prior studies have demonstrated the great potential of BCL as a potent antimicrobial, and it has significant effects on disruption of biofilm structure, inactivation of virulence factors such as quorum sensing, bacteriocins etc. against various pathogens (Cai et al. 2016, Luo et al. 2016b, Vijayakumar et al. 2021).

In search of a novel strategy, we propose that BCL may have an improved antimicrobial action when combined with an antibiotic that is currently a viable therapy option. This strategy should prove useful in (i) treating antibiotic-resistant infections, (ii) limiting the emergence of antibiotic resistance, and (iii) managing the side effects of antibiotics, such as toxicity, due to reduced chemotherapeutic concentrations.

In this study, a series of clinical XDR and PDR *A. baumannii* isolates was first subjected to detailed microbiological characterization with the aim of profiling their antibiotic resistance phenotypically and genotypically. Next, the combined antimicrobial and antibiofilm activity of meropenem (MEM) and BCL against these isolates was assessed by using in vitro experiments. Last, in an *in silico* setting, the ability of BCL to bind *A. baumannii* OXAs (oxacillinase type of beta-lactamases) and PBPs (Penicillin binding protein) was predicted based on molecular docking calculations.

## Materials and methods

### Materials

Mueller–Hinton broth (MHB) (cat. no: 1.10293.0500), blood agar base (cat. no: 1.10886.0500), and Mueller–Hinton agar (MHA) (cat no: 1.05437.0500) were purchased from Merck (Germany) and were used for cultivating the bacteria and/or for the assays. BCL (P.N:465119) and MEM (P.N: 32 460) were purchased from Sigma–Aldrich (USA) and stored according to the manufacturer’s recommendations. BCL was dissolved in dimethyl sulfoxide (final concentration did not exceed 5% in all experiments), and MEM was dissolved in sterile distilled water. Glycerol supplemented brain heart infusion broth (cat. no: M210-500G) was purchased from Himedia (India) and used for storage of bacterial isolates. BD-Crystal® Enteric/Non-fermenter Identification kit was used for confirmation of pure isolation of isolates. *Escherichia coli* ATCC 25922 was used as the bacterial strain sensitive to imipenem in the modified Hodge test (MHT). All solutions’ pH values were checked and adjusted routinely after preparation of all solutions and before usage in the assays.

### Bacterial isolates and characterization

Bacterial strains used in this study were isolated from Çukurova University Hospital in Turkey. *Acinetobacter baumannii* strains were identified at the species level by matrix-assisted laser desorption/ionization-time-of-flight mass spectrometry (MALDI-TOF MS), and initial antibiotic resistance patterns were determined by the MicroScan WalkAway plus System (Beckman Coulter, USA).

### Antibiotic resistance profiling and confirmation of carbapenem resistance

The antibiotic resistance profiles of the selected strains were established by measuring the limit values for various antibiotics with the Kirby–Bauer disk diffusion method according to the EUCAST criteria (EUCAST 2015). Resistance characteristics of the strains were evaluated according to the EUCAST guidelines by measuring zone diameters after 18–20 h of incubation (EUCAST 2015).

Strains that were found to be resistant to carbapenems were further evaluated with MHT to confirm carbapenem resistance as described previously (Girlich et al. 2012).

### Genotypic analysis of antibiotic resistance

Nucleic acid extraction of the strains was achieved by boiling and applying the freeze-thawing method without using any chemicals (Chen et al. 2020). Then polymerase chain reaction (PCR) targeting the *bla*_OXA-51_ gene was performed to confirm the identification of the isolates as carbapenemase producer *A. baumannii*. Isolates were searched for antibiotic resistance genes using multiplex PCR panels to detect Ambler class *bla* genes and mobile genetic elements. Primers were selected to amplify the following *bla* gene groups: class A, *bla*_TEM_, *bla*_SHV_, *bla*_CTX-M_, *bla*_VEB_, *bla*_PER_, *bla*_KPC_, and *bla*_GES_; class B, *bla*_IMP_, *bla*_VIM_, *bla*_GIM_, *bla*_SPM_, *bla*_SIM_, and *bla*_NDM_; and class D, *bla*_OXA-23-like_, *bla*_OXA-24-like_, and *bla*_OXA-51-like_ as described previously (Dallenne et al. 2010). For screening colistin resistance, the mcr-1 gene was screened by PCR method by using primers as described previously (Liu et al. 2016). All PCR assays were carried out by using Thermo Scientific Taq green master mix in Applied Biosystems 2720 thermocycler (Thermo Fisher Scientific, CA, USA). Positive and negative controls were included in all PCR assays. Resistance genes pre-detected by PCR were further examined by DNA sequence analysis, and the specific sequences for the resistance gene were identified based on closest Blast matches from the NCBI database (https://blast.ncbi.nlm.nih.gov/Blast.cgi). Sequencing reactions were carried out by using the same primer sets and BigDye™ Terminator v3.1 Cycle Sequencing Kit (Applied Biosystems, Foster City, CA, USA) in an automated Sanger sequencer (ABI 310 Genetic analyzer; Applied Biosystems, Foster City, CA, USA).

### Determination of MICs

Broth micro dilution assay was conducted to determine the minimum inhibitory concentrations (MICs) of MEM and BCL against the *A. baumannii* isolates. Briefly, MEM and BCL stock solutions were prepared and adjusted to 1 mg/ml. Suspensions of bacterial samples were prepared to 0.5 McFarland turbidity standard and then diluted 100-fold using saline to adjust the final bacterial cell numbers to 1 × 10^6^ CFU/ml. A volume of 50 µl of MHB was added to each well of a microplate. Next, 50 µl of test solution was added and diluted serially to wells containing MHB, except the controls. Finally, 50 µl of bacterial suspension was added to all wells, except the negative control. The microplates were incubated at 37°C for 24 h under aerobic conditions. MIC values were determined as the lowest concentration of the antimicrobial agent where the growth of bacteria was inhibited. Results were interpreted according to the EUCAST guidelines (EUCAST 2015).

### Synergy testing: checkerboard and time-kill assays

The putative antimicrobial synergy between MEM and BCL was analyzed by performing a checkerboard assay (Sopirala et al. 2010), which normally depends on testing the susceptibility of strains to different combinations of multiple agents at varying doses. The starting concentrations of the agents were 4 × MIC and were diluted until 1/32 × MIC for both for MEM and BCL, respectively. The combined effect of the agents was evaluated by calculating the total fractional inhibitory concentration index (ΣFICI).

Time-kill kinetic assays were performed only for strains, which displayed highest MICs for MEM, lowest MICs for BCL, and were found to be “synergistic” in the checkerboard assay (Barry et al. 1999). Briefly, MHB with agents alone and in combinations were inoculated with an aliquot of the strain with a concentration of ∼10^5^ CFU/ml. MEM and BCL were evaluated at 0.5 × MIC, alone and/or combined in addition to control, which was 0.1% DMSO. Samples were taken at 0, 2, 4, 8, 12, and 24 h followed by serial dilution in saline and plating on MHA at an amount of 50 μl. Plates were incubated at 37°C for 24 h, for CFU counting. Reduction in two or more logarithmic units between the combined and the most active agent alone after 24 h was considered as synergistic.

### Biofilm inhibition assay

The biofilm inhibitory potentials of MEM and BCL either alone or in combination with each other against *A. baumannii* were investigated by using MIC and sub-MIC (MIC/2 or MIC/4) concentrations of the agents in a 96-well plate. Control samples, which were prepared with no added drug, were also included in the assays. Firstly, 96-well plates containing bacterial samples at a final concentration of 1 × 10^6^ cfu/ml and varying concentrations of the agents were incubated for 24 h at 37°C to generate biofilms. After biofilm production, the wells were gently washed three times with saline solution to get rid of planktonic cells and to keep only the cells that adhered onto the wells’ inner surface. To dye the biofilm layers, 125 µl of 1% (w/v) crystal violet solution was added into every well and incubated for 30 min, followed by cleaning with saline solution. Finally, previously prepared 95% (v/v) ethanol was added to every well and incubated for 30 min to decolorize the bacteria and to stain the biofilms. Lastly, the absorbance values of the solutions were measured at 570 nm on a Varioskan Flash Multi Detection Microplate Reader (Thermo Fisher Scientific, USA). The obtained absorbance data were analyzed by quantitatively estimating the biofilm inhibition, in which the control samples were taken as standards. The results were presented as % inhibition of biofilm formation.

### Protein–ligand docking and interaction profiling

The 3D conformer of BCL (compound ID: 5281605) in SDF format was retrieved from the PubChem open chemistry database available at https://pubchem.ncbi.nlm.nih.gov/ (Kim et al. 2021). The crystal structures of (i) *A. baumannii* OXA-23 in complex with MEM (PDB ID: 4JF4), (ii) *A. baumannii* OXA-51 (K83D/I129L variant) in complex with doripenem (PDB ID: 5L2F), and (iii) *A. baumannii* PBP1a in complex with imipenem (PDB ID: 3UDX) were downloaded from the RCSB Protein Data Bank available at https://www.rcsb.org/ (Burley et al. 2021). Hydrogen coordinates and appropriate protonation states were assigned to PBP1a and the OXAs of interest by using the Protoss hydrogen prediction tool available at https://proteins.plus/ (Ertl and Schuffenhauer 2009, Bietz et al. 2014). BCL was docked in the presence of structurally relevant water molecules onto the *A. baumannii* proteins by using the JAMDA molecular docking tool available at https://proteins.plus/ (Schellhammer and Rarey 2007, Henzler et al. 2014, Flachsenberg et al. 2020). Each binding site was defined by the *bona fide* carbapenem ligand, with a site radius of 6.5 Å. Molecular docking was executed with medium precision. Favorable non-covalent interactions between BCL and the *A. baumannii* proteins were computed by using Discovery Studio Visualizer, v16.1.0 (Dassault Systèmes BIOVIA Corp., San Diego, CA, USA).

### Statistical analysis

Data were statistically analyzed and visualized by using the GraphPad Prism Software, LLC., Version 9.2.0 (GraphPad Software Inc., San Diego, CA, USA). One-way ANOVA followed by Sidak’s multiple comparisons test was employed to analyze the reductions in MIC values. χ^2^-test was used to compare biofilm inhibition percentages among different concentrations. *P-*values of < .05 were regarded significant, and asterisks were used to indicate significant differences between different samples/sample sets.

## Results

### Antibiotic resistance profiling demonstrates XDR and PDR phenotype and genetic background of the resistance

A total of 15 clinical isolates were selected for this study. The isolates were collected from internal medicine service and cultivated from different clinical specimens including aspirate (*n* = 8), blood (*n* = 5), wound (*n* = 1), and sputum (*n* = 1). Isolates were collected from nine males and six females in an age range between 41 and 76 with a mean age of 66.47 ± 2.34 years. Clinical data of the isolates, their genes responsible for antibiotic resistance, and their antimicrobial responses to different antibiotics are shown in Table 1. With respect to carbapenem resistance, all isolates were discovered to be resistant to both imipenem and MEM in disk diffusion assays. The results of the MHT further confirmed the carbapenem resistance for the entire set of isolates.

**Table 1.** Clinical information and antibiotic susceptibility of *A. baumannii* isolates.

				Type of tested antibiotics^a^									
Bacterial strains	Age	Gender	Type of specimen	FEP	CAZ	TPZ	GEN	AMK	MEM	IPM	CT	Resistance category^b^	Resistance genes
AB1	41	M	Aspirate	R	R	R	R	R	R	R	R	PDR	OXA-51 + TEM + OXA-48 + OXA-23
AB2	58	M	Blood	R	R	R	R	R	R	R	R	PDR	OXA-51 + TEM + OXA-48 + OXA-23
AB3	69	F	Aspirate	R	S	R	R	R	R	R	R	PDR	OXA-51 + OXA-48 + OXA-23 + VIM
AB4	69	M	Aspirate	R	R	R	R	R	R	R	R	PDR	OXA-51 + TEM + OXA-23 + VIM
AB5	60	M	Blood	R	R	R	R	R	R	R	R	PDR	OXA-51 + TEM + OXA-48 + OXA-23 + VIM
AB6	67	M	Aspirate	R	R	R	R	R	R	R	R	PDR	OXA-51 + TEM + OXA-48 + OXA-23 + IMP + VIM
AB7	76	F	Aspirate	R	R	R	R	R	R	R	R	PDR	OXA-51 + TEM + OXA-48 + OXA-23
AB8	76	F	Blood	R	R	R	R	R	R	R	R	PDR	OXA-51 + TEM + OXA-23 + VIM
AB9	73	F	Aspirate	R	R	R	R	R	R	R	S	XDR	OXA-51 + OXA-48 + OXA-23 + VIM
AB10	65	M	Aspirate	R	R	R	R	R	R	R	S	XDR	OXA-51 + TEM + OXA-48 + OXA-23 + VIM
AB11	62	M	Sputum	R	R	R	R	R	R	R	S	XDR	OXA-51 + OXA-23
AB12	73	F	Wound	R	R	R	R	R	R	R	S	XDR	OXA-51 + OXA-23 + VIM
AB13	72	F	Aspirate	R	R	R	R	R	R	R	S	XDR	OXA-51 + TEM + OXA-23 + VIM
AB14	63	M	Blood	R	R	R	R	R	R	R	S	XDR	OXA-51 + OXA-23
AB15	73	M	Blood	R	R	R	R	R	R	R	S	XDR	OXA-51 + TEM + OXA-23

a FEP: Cefepime, CAZ: Ceftazidime, TPZ: Piperacillin/Tazobactam, GEN: Gentamycin, AMK: Amikacin, MEM: Meropenem, IPM: Imipenem, and CT: Colistin.

b The isolates were categorized as multidrug-resistant (MDR, resistance to at least three classes of antimicrobials), extensively drug resistant (XDR, MDR plus resistance to carbapenems), and pan-drug-resistant (PDR, XDR plus resistance to polymyxins) (Karaiskos and Giamarellou 2014).

### Combination of MEM and BCL demonstrates synergism or additive antibacterial interactions against all isolates

The MIC values of *A. baumannii* isolates against MEM and BCL were investigated by broth microdilution assay. As depicted in Table 2, the results of the assays revealed that *A. baumannii* isolates had a range of MIC values between 15.60–125 mg/l and 7.80–31.25 mg/l against MEM and BCL, respectively.

**Table 2.** Summary of antimicrobial susceptibility and combined effects of MEM and BCL.

Isolates	Resistance category	MICs^a^		MICs^a^ in combination		FICI		∑FICI	Interpretation^b^
		MEM	BCL	MEM	BCL	MEM	BCL		
AB1	PDR	62.50	15.60	15.60	0.97	0.25	0.06	0.31	Synergy
AB2	PDR	125.00	31.25	3.90	15.62	0.03	0.50	0.53	Additive
AB3	PDR	125.00	15.60	15.60	1.95	0.12	0.13	0.25	Synergy
AB4	PDR	15.60	31.25	3.90	7.80	0.25	0.25	0.50	Synergy
AB5	PDR	15.60	31.25	3.90	15.60	0.25	0.50	0.75	Additive
AB6	PDR	31.25	15.60	3.90	1.95	0.12	0.13	0.25	Synergy
AB7	PDR	31.25	15.60	3.90	0.97	0.12	0.06	0.19	Synergy
AB8	PDR	31.25	15.60	15.60	1.95	0.50	0.13	0.62	Additive
AB9	XDR	31.25	15.60	7.80	7.80	0.25	0.50	0.75	Additive
AB10	XDR	31.25	15.60	7.80	7.80	0.25	0.50	0.75	Additive
AB11	XDR	15.60	15.60	3.90	7.80	0.25	0.50	0.75	Additive
AB12	XDR	15.60	15.60	3.90	0.97	0.25	0.06	0.31	Synergy
AB13	XDR	15.60	7.80	1.90	1.95	0.12	0.25	0.37	Synergy
AB14	XDR	15.60	15.60	3.90	1.90	0.25	0.12	0.37	Synergy
AB15	XDR	15.60	15.60	1.95	3.90	0.13	0.25	0.38	Synergy
Mean		38.53	18.21	6.50	5.26	0.21	0.26	0.47	

The results were given as mg/l.

a The results are given in mg/l.

b ΣFICI ≤ 0.5—synergic effect, 0.5 < ΣFICI ≤ 4—additive effect, and ΣFICI > 4—antagonistic effect.

**Figure 1.**
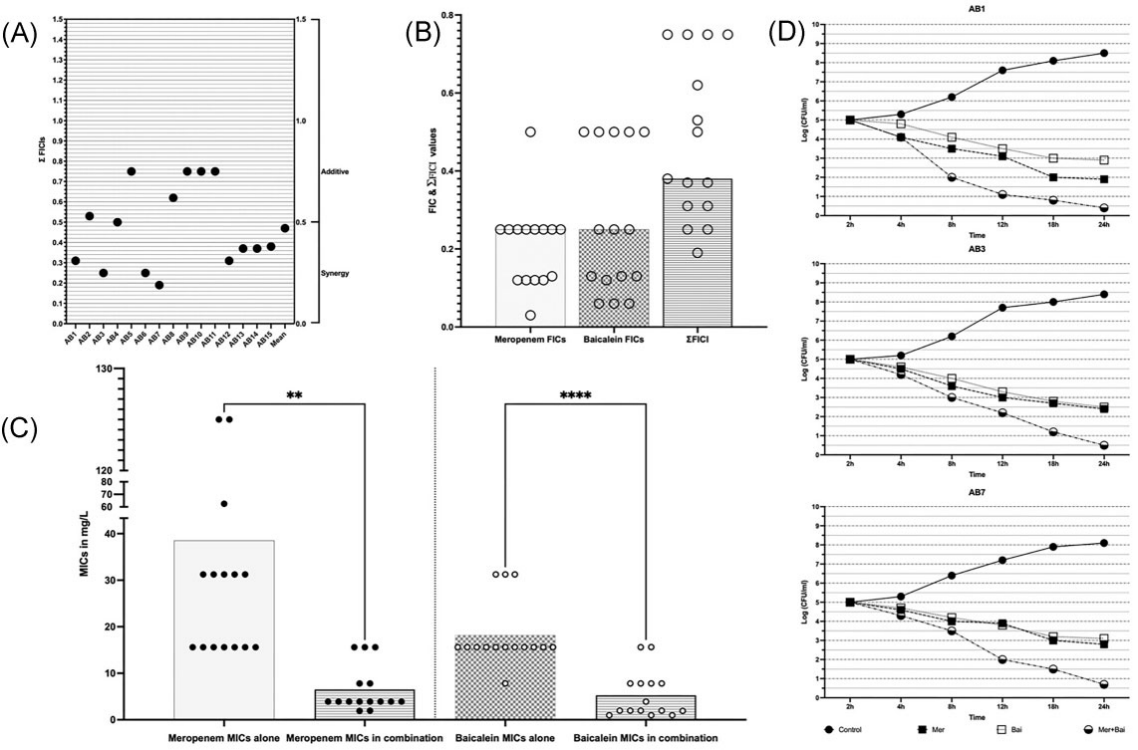
Synergistic interactions of MEM and BCL against XDR and PDR *A. baumannii* isolates. (A) Checkerboard synergy assay results of individual isolates according to calculated EFIC (Total fractional inhibitory concentration index) values. (B) Distribution of FIC (Fractional inhibitory concentration index) and EFIC values of agents against isolates, where FIC is calculated by dividing MICs of agents in combination to MICs alone and EFIC is the sum of FICs of agents in combination. (C) Changes of MICs of agents when in combination vs. alone. Meropenem’s mean of MICs is reduced from 38.53 to 7.54 (≈5-fold reduction) in combination (*P* = .008). Baicalein’s mean of MICs is reduced from 23.94 to 7.201(≈3-fold reduction) in combination (*P* = .0001). (D) Time-kill assay results of strains AB1, AB3, and AB7. Double-fold logarithmic reductions (1.9–0.4, 2.4–0.5, and 2.8–0.7 CFUs when MEM was alone and when in combination for AB1, AB3, and AB7, respectively) were observed between the combination of agents and MEM alone after 24 h indicating synergism.

The combined inhibitory actions of MEM and BCL on *A. baumannii* isolates were evaluated by a checkerboard assay firstly. This assay demonstrated that MEM and BCL had synergistic/additive effects on all isolates. Indifference or antagonism was not observed for any isolate, suggesting a productive alliance between the two agents. Synergistic interactions were observed for 9 out of 15 *A. baumannii* isolates (46.66%), and additive interactions were detected for 6 out of 15 *A. baumannii* isolates (53.4%). Synergism was applicable to 5 out of 8 PDR *A. baumannii* isolates, while 4 out of 7 XDR *A. baumannii* isolates were affected by synergism between the agents. Detailed MICs of agents alone, MICs of agents in combination and FICI values are given in Table 2 and Fig. 1.

To confirm synergism between MEM and BCL a time-kill assay was performed for three representative strains namely, AB1, AB3, and AB7. These three isolates are selected because of being (i) PDR, (ii) highly resistant to MEM, (iii) susceptible to BCL, and (iv) found to be synergistic in checkerboard assay. Synergism was observed in three tested strains confirming the results of checkerboard assay. Detailed results of time-kill assay are given in Fig. 1.

### MEM and BCL combination inhibit biofilm formation significantly

**Figure 2.**
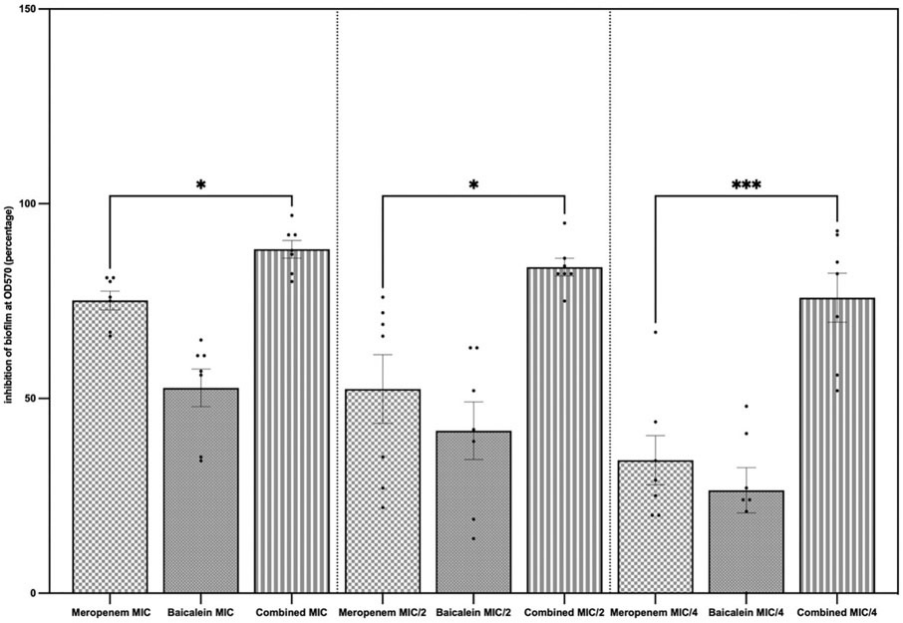
Standalone and combined antibiofilm activities of MEM and BCL. Biofilm inhibition percentages were calculated by measuring crystal violet absorbance spectrophotometrically at lambda max 570 nm and comparing with control. The results are demonstrated for concentrations of agents at MIC, MIC/2, and MIC/4. Highest inhibition was observed when the agents were combined at MIC (mean = 88.29%), followed by combined at MIC/2 (mean = 83.71%) and combined at MIC/4 (mean = 75.86%). Mean biofilm inhibition of MEM at MIC was 75.14%. When the agents were combined, biofilm inhibition was significantly higher when compared to MEM alone (*P* = .0471 at MIC, *P* = .0406 at MIC/2, and *P* = .004 at MIC/4).

The effect of MEM and BCL on biofilm formation, which is an important pathogenic determinant of *A. baumannii*, was studied by conducting a biofilm inhibition assay. MEM and BCL inhibited biofilm formation both separately and combined in a concentration-dependent manner. It was revealed that combining the antimicrobials at concentrations of MIC/2 and MIC/4 exhibited significantly higher inhibition against biofilm formation by *A. baumannii* compared to the standalone MIC concentrations (*P* < .0001). Biofilm inhibition assay results in detailed percentages are given in Table 3, and the inhibitory effects of MEM, BCL, and their combinations on biofilm formation are illustrated in Fig. 2.

**Table 3.** Biofilm inhibition assay results in percentages.

	MEM			BCL			Combination		
	MIC	MIC/2	MIC/4	MIC	MIC/2	MIC/4	MIC	MIC/2	MIC/4
AB3	76	69	67	57	42	27	82	82	92
AB4	80	72	34	65	63	48	87	86	93
AB6	75	66	44	61	63	41	80	82	85
AB12	81	76	29	56	52	0	92	82	71
AB13	66	35	25	61	39	24	97	95	82
AB14	67	27	20	34	19	24	92	84	52
AB15	81	22	20	35	14	21	88	75	56

### *In silico* studies shed light to OXA and PBP binding potential of BCL

**Figure 3.**
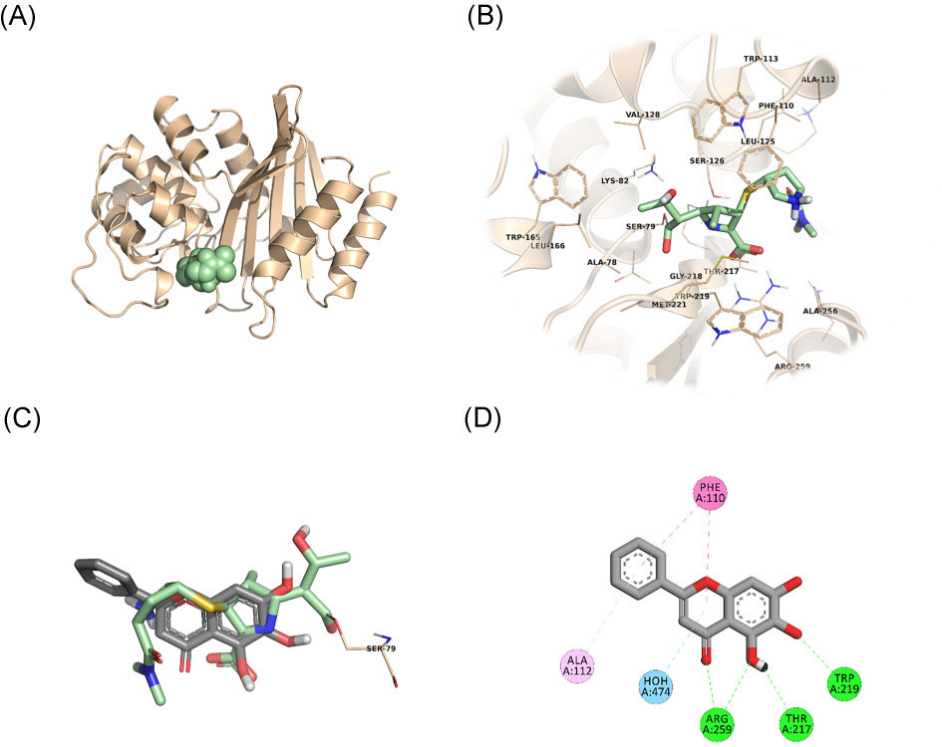
OXA-23–BCL docking and interaction profiling. (A) Ribbon representation of the *A. baumannii* OXA-23 monomer (wheat), with the bound MEM molecule shown as pale green spheres (PDB ID: 4JF4). (B) Zoomed view of MEM (pale green sticks) in the active-site cleft of OXA-23. The side chains of residues within 4 Å of MEM are shown in line representation. (C) Close-up view of the superposed structures of MEM (pale green sticks) and the docked BCL molecule (gray sticks) at the active site of OXA-23. Ser79, which forms an acyl linkage with MEM, is shown as lines. (D) Favorable non-covalent interactions predicted to occur between OXA-23 and BCL. Lime green dashed lines correspond to conventional H-bonds [within 3.4 Å (strong) or 3.8 Å (weak)], aqua blue dashed lines correspond to water H-bonds, magenta dashed lines correspond to π–π stacking interactions (within 6.0 Å), and lilac dashed lines correspond to π–alkyl interactions (within 5.5 Å). (A), (B), and (C) were rendered by using the PyMOL Molecular Graphics System, v1.8 (Schrödinger LLC, Portland, OR, USA). (D) was rendered by using Discovery Studio Visualizer, v16.1.0 (Dassault Systèmes BIOVIA Corp., San Diego, CA, USA). Non-polar hydrogen atoms are not shown for clarity.

**Figure 4.**
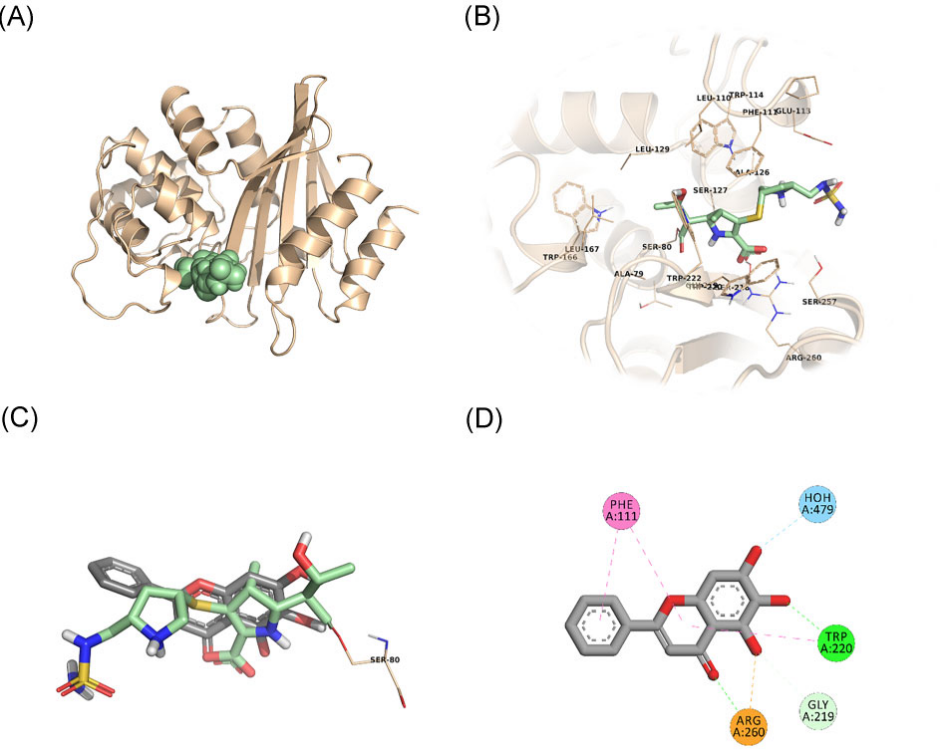
OXA-51–BCL docking and interaction profiling. (A) Ribbon representation of the *A. baumannii* OXA-51 (K83D/I129L variant) monomer (wheat), with the bound doripenem molecule shown as pale green spheres (PDB ID: 5L2F). (B) Zoomed view of doripenem (pale green sticks) in the active-site cleft of OXA-51. The side chains of residues within 4 Å of doripenem are shown in line representation. (C) Close-up view of the superposed structures of doripenem (pale green sticks) and the docked BCL molecule (gray sticks) at the active site of OXA-51. Ser80, which forms an acyl linkage with doripenem, is shown as lines. (D) Favorable non-covalent interactions predicted to occur between OXA-51 and BCL. Orange dashed lines correspond to electrostatic interactions (within 5.0 Å), lime green dashed lines correspond to conventional H-bonds [within 3.4 Å (strong) or 3.8 Å (weak)], seafoam green dashed lines correspond to carbon H-bonds (within 3.8 Å), aqua blue dashed lines correspond to water H-bonds, and magenta dashed lines correspond to π–π stacking interactions (within 6.0 Å). (A), (B), and (C) were rendered by using the PyMOL Molecular Graphics System, v1.8 (Schrödinger LLC, Portland, OR, USA). (D) was rendered by using Discovery Studio Visualizer, v16.1.0 (Dassault Systèmes BIOVIA Corp., San Diego, CA, USA). Non-polar hydrogen atoms are not shown for clarity.

**Figure 5.**
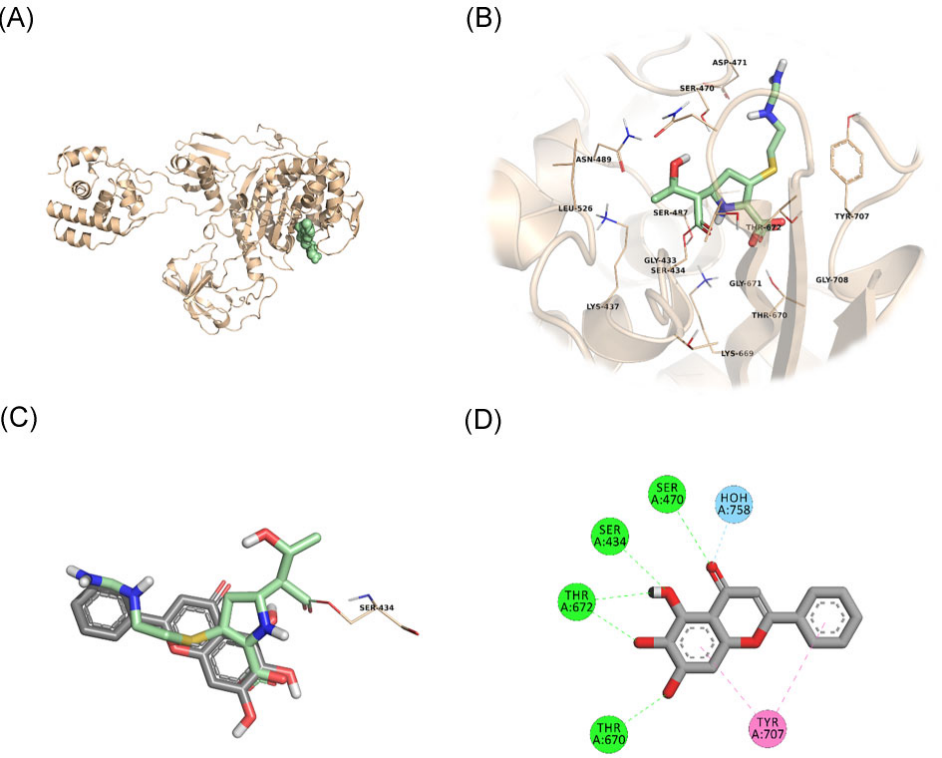
PBP1a–BCL docking and interaction profiling. (A) Ribbon representation of the *A. baumannii* PBP1a monomer (wheat), with the bound imipenem molecule shown as pale green spheres (PDB ID: 3UDX). (B) Zoomed view of imipenem (pale green sticks) in the active-site cleft of PBP1a. The side chains of residues within 4 Å of imipenem are shown in line representation. (C) Close-up view of the superposed structures of imipenem (pale green sticks) and the docked BCL molecule (gray sticks) at the active site of PBP1a. Ser434, which forms an acyl linkage with imipenem, is shown as lines. (D) Favorable non-covalent interactions predicted to occur between PBP1a and BCL. Lime green dashed lines correspond to conventional H-bonds [within 3.4 Å (strong) or 3.8 Å (weak)], aqua blue dashed lines correspond to water H-bonds, and magenta dashed lines correspond to π–π stacking interactions (within 6.0 Å). (A), (B), and (C) were rendered by using the PyMOL Molecular Graphics System, v1.8 (Schrödinger LLC, Portland, OR, USA). (D) was rendered by using Discovery Studio Visualizer, v16.1.0 (Dassault Systèmes BIOVIA Corp., San Diego, CA, USA). Non-polar hydrogen atoms are not shown for clarity.

In an attempt to predict the inhibitory activity of BCL against resistance-related *A. baumannii* targets, BCL was docked into the active-site clefts of OXA-23, OXA-51, and PBP1a in a “closed” (i.e. carbapenem-bound) conformation. The finest docking solutions were selected according to computed JAMDA scores as well as additional structural criteria already described for *bona fide* carbapenem ligands (Han et al. 2011, Smith et al. 2013, June et al. 2016). The results of our molecular docking simulations revealed that BCL could be accommodated well in the active-site clefts of OXA-23 (JAMDA score: –2.24887) and OXA-51 (JAMDA score: –2.24221). In the predicted OXA-23–BCL complex (Fig. 3), the ligand was demonstrated to form hydrogen-bonding interactions with Thr217, Trp219, and Arg259 through its hydroxyl groups (namely, C5–OH and C6–OH) on the benzene ring (A-ring). Arg259 was able to form an additional hydrogen bond with the carbonyl oxygen on the heterocyclic pyran ring (C-ring) of BCL. Phe110 was found to be engaged in aromatic stacking interactions with the C-ring as well as the phenyl substitution at position 2 (B-ring). A similar interaction pattern was also observed for the predicted OXA-51–BCL complex (Fig. 4). Gly219 was found to form a carbon–hydrogen bond with the C5–OH group of the ligand. Trp220 (which corresponds to Trp219 in OXA-23) was able to form a hydrogen bond with the C6–OH group and an aromatic stacking interaction with the C-ring. The C5–OH group in its deprotonated form (calculated p*K*_a_: 8.35) appeared to be salt bridged to Arg260 (which corresponds to Arg259 in OXA-23). Arg260 could also form a hydrogen bond with the carbonyl oxygen on the C-ring. The C and B rings were further stabilized by aromatic stacking interactions with Phe111 (which corresponds to Phe110 in OXA-23). Overall, these findings support the notion that BCL could possibly exert its synergistic effect by competing with MEM for binding at the active sites of the two most prevalent OXAs in the clinical *A. baumannii* isolates of question, thereby inhibiting MEM hydrolysis and increasing the effective concentrations of MEM in the *in vitro* setting. An alternative, or most likely an additional, synergistic role played by BCL could be its binding to PBPs in *A. baumannii*, thus inhibiting cell wall formation and potentiating the antibacterial action of MEM. Our docking calculations revealed that BCL could be housed well in the active-site cleft of PBP1a (JAMDA score: –2.11966) and that it could establish hydrogen-bonding interactions with Ser434, Thr670, and Thr672 through its hydroxyl groups (C5, C6, and C7) on the A-ring (Fig. 5). An additional hydrogen bond could be formed between the carbonyl oxygen on the C-ring and Ser470. Tyr707 was found to be an important residue in further stabilizing BCL via aromatic stacking interactions with the A and B rings.

## Discussion

In this report, successful sensitization of XDR and PDR *A. baumannii* isolates by combining MEM with BCL was achieved, and synergism between the agents was demonstrated in terms of both antibacterial and antibiofilm actions. Furthermore, we provided structural and mechanistic insights into how this synergism might work.

The isolates used in this study were eight PDR and seven XDR *A. baumannii* strains, which were resistant to all tested antibiotics (except that the XDR strains were only susceptible to colistin) representing major antibiotic classes. The carbapenem resistance of all isolates was further confirmed by MHT. Genotypical analysis demonstrated a wide arsenal of resistance genes, which were predominantly OXA-51, OXA-23, followed by OXA-48, OXA-23, TEM, VIM, and IMP, and these results were correlated with the susceptibility profiles of the isolates.

Here, we tested the performance of a highly promising compound, BCL in combination with MEM in an effort to overcome antibiotic resistance in a subset of *A. baumannii* clinical isolates. When combined with BCL, the MIC values of MEM were significantly reduced. In fact, *in vitro* test concentrations are very instructive for us and can readily be translated to *in vivo* drug doses, highlighting the additional potential benefits of our combination strategy, such as decreasing the toxicity and/or managing the adverse effects of MEM (Baldwin et al. 2008, Liu et al. 2020). It is worth noting that BCL has almost no toxicity to human cells as reported in earlier studies (Dinda et al. 2017). Furthermore, reaching to concentrations as low as 0.97 mg/l for BCL in combination is promising when evaluated with peak concentrations achieved in human serum (Li et al. 2021).

In our setting, a significant reduction in MEM concentrations was achieved in killing of the isolates where MEM–BCL combination showed synergism or additive action against all isolates. Moreover, the mean MIC value of BCL against XDR/PDR isolates was 18.21 mg/l [5.26 mg/l (0.97 mg/l lowest) in combination], which was reasonably low for a natural flavonoid. To compare, in a similar study focusing on carbapenem resistant *A. baumannii* isolates, quercetin, which is a well-known natural flavonoid for its antimicrobial activity demonstrated MICs around 64 mg/l against tested isolates (Pal and Tripathi 2019).

In addition to generating a potent antimicrobial synergism, BCL and MEM are cooperating well against the biofilm structure of the pathogen as well. Biofilm inhibition percentages of the agents were significantly higher in combination compared to standalone applications especially at lower concentrations. Exopolysaccharide matrices produced by *A. baumannii* make the bacteria more resistant to external stressors including antibiotics mainly by making it harder for the antibiotic molecule to reach the target cell (Harding et al. 2018). In the relevant scientific literature, there are only a few studies evaluating the anti-biofilm activity of BCL against various pathogens. These reports suggest that BCL holds the potential to inhibit biofilm formation, destroy biofilms, increase the permeability of various antibiotics, and block quorum sensing in selected pathogens (Chen et al. 2016, Shirley et al. 2017, Vijayakumar et al. 2021). Although most of these studies involve Gram-positive bacteria or yeasts, our results are consistent with them and considered novel as we provide antibiofilm activity data for BCL against *A. baumannii* (XDR and PDR) for the first time in this investigation. The mechanism underlying the anti-biofilm property of BCL warrants further tests, but recent studies have suggested different modes of action pertaining to diverse agents in well-known pathogens (Chen et al. 2016, Luo et al. 2016a, Najarzadeh et al. 2019).

Natural products have historically played a significant role in drug discovery. In many instances, BCL was found to restore/enhance the *in vitro* antibacterial activity of MEM against the *A. baumannii* clinical isolates in question. The mechanism underlying this synergistic effect could be interpreted as the putative ability of BCL to inhibit MEM-hydrolyzing β-lactamases, such as OXAs, in *A. baumannii*, and/or prevent *A. baumannii* from constructing a cell wall by binding to peptidoglycan-synthesizing PBPs, in a similar fashion to MEM. Although additive (and even indifferent) interactions are also able to minimize antimicrobial resistance development in bacteria (Noel et al. 2021), the non-specific mechanisms underlying additivity may be difficult to ascertain because of a wide range of cellular targets as well as strain-dependent genetic and biochemical networks.

Using structural information and kinetic data obtained for the OXA-51 I129L variant and other variants from clinical *A. baumannii* isolates, June *et al*. have proposed that OXA-51-like enzymes are only one to two substitutions away from acquiring kinetic properties similar to or identical to those observed for OXA-23 (June et al. 2016). Here, using computational studies, we show that BCL holds the potential to inhibit OXA-23 and OXA-51, two major β-lactamases present in the entire set of clinical *A. baumannii* isolates included in this work. Owing to its trihydroxychromenone structure and the attached phenyl ring, BCL may also bind to *A. baumannii* PBP1a and ideally render this cell wall synthesis enzyme inactive. Unlike high-molecular-weight (HMW) class B PBPs that perform only transpeptidase functions, HMW class A PBPs (e.g. PBP1a and PBP1b) are bifunctional enzymes performing both transpeptidase and transglycosylase functions (Ghuysen 1991). Although PBPs probably make a slight contribution to antibiotic resistance in bacteria, identifying compounds that bind PBPs will eventually pave the way for the structure-based design of more effective antibiotics. It should be mentioned here, however, that our *in silico* findings are yet to be confirmed by further *in vitro* and/or *in vivo* studies.

Our research has several limitations to note. First and foremost, a larger set of isolates including standard strains may be required to further validate the results. Next, our findings concern the *in vitro* activity of the investigated antimicrobial combinations and should be understood with the knowledge that *in vitro* susceptibility data are not the only factor to consider when determining the most optimal antibiotic treatment for patients. Last, because this is a single-center study, it may not accurately reflect the susceptibility of strains with similar characteristics identified in elsewhere.

To conclude, our findings provide an important basis for the development of viable alternative regimens to treat patients suffering from XDR/PDR *A. baumannii* infections. Future studies are suggested to elucidate other likely interactions and mechanisms behind the combinatory actions of BCL and MEM. Also, further *in vivo* studies are required to validate the clinical use and effectiveness of this novel treatment strategy.
